# The effects of experimental, meteorological, and physiological factors on short-term repeated pulse wave velocity measurements, and measurement difficulties: A randomized crossover study with two devices

**DOI:** 10.3389/fcvm.2022.993971

**Published:** 2023-01-11

**Authors:** Mario Podrug, Borna Šunjić, Anamarija Bekavac, Pjero Koren, Varja Đogaš, Ivana Mudnić, Mladen Boban, Ana Jerončić

**Affiliations:** ^1^Laboratory of Vascular Aging, University of Split School of Medicine, Split, Croatia; ^2^University Department of Health Studies, University of Split, Split, Croatia; ^3^PhD Study Programme, University of Split School of Medicine, Split, Croatia; ^4^Department of Research in Biomedicine and Health, University of Split School of Medicine, Split, Croatia; ^5^Department of Psychological Medicine, University of Split School of Medicine, Split, Croatia; ^6^Department of Basic and Clinical Pharmacology, University of Split School of Medicine, Split, Croatia

**Keywords:** carotid-femoral pulse wave velocity, pulse wave velocity, within subject variation, predictors, meteorological conditions, experimental conditions, measurement error, measurement difficulty

## Abstract

**Background:**

Large longitudinal studies with repeated pulse wave velocity (PWV) measurements, a direct measure of arterial stiffness, are required to realize the full potential of arterial stiffness in clinical practice. To facilitate such studies it is important to increase the power of a study by reducing within-subject variability of PWV, and to ease the use of a PWV device in clinical settings by minimizing PWV measurement difficulties.

**Methods:**

We systematically investigated experimental setting and meteorological conditions, as well as physiological factors and participant characteristics, to determine whether and to what extent they affected: between- and within-subjects variability of PWV recordings, and measurement difficulties of a particular device. We conducted a 2-week longitudinal block-randomized cross-over study with two blinded observers and two commonly used devices: applanation tonometry SphygmoCor CvMS and oscillometric Arteriograph to assess carotid-femoral (cfPWV) or aortic (PWVao) PWV, respectively. Our sample had uniform and wide-spread distribution of age, blood pressures, hypertensive status and BMI. Each participant (*N* = 35) was recorded 12 times over 3 visiting days, 7 days apart. On each day, recordings were made twice in the morning (7–10 a.m.) and afternoon (16–18 p.m.). Data were analyzed using multilevel mixed-effects models, separately for each device.

**Results:**

In addition to age and mean arterial pressure (MAP) that strongly affected both cfPWV and PWVao, other significant factors appeared to indicate a measurement approach. cfPWV as a more direct measure of arterial stiffness was additionally affected by hypertension status, outdoor temperature, interaction of MAP with outdoor temperature and the order of visit, with MAP within-subject variability contributing on average 0.27 m/s to difference in repeated measurements at 5^°^C and 0.004 m/s at 25^°^C. PWVao measurements derived at a single brachial site were more dependent on age than cfPWV and also depended on personal characteristics such as height and sex, and heart rate; with within-subject MAP variability adding on average 0.23 m/s to the difference in repeated measures. We also found that female sex significantly increased, and recording in afternoon vs. morning significantly decreased measurement difficulties of both devices.

**Conclusion:**

We identified factors affecting PWV recordings and measurement-difficulties and propose how to improve PWV measuring protocols.

## 1. Introduction

Arterial stiffness is a phenomenon associated with vascular aging that refers to loss of arterial compliance or changes in vessel wall properties (1). Arterial stiffness increases with age and with prolonged exposure to risk factors that accelerate this process (2, 3). Numerous studies have found that increased arterial stiffness is associated with an increased risk of a first or recurrent major CVD event, independent of traditional risk factors, in both disease-specific and population-based samples (4–6). In addition, arterial stiffness has been shown to improve reclassification of patients at intermediate risk for cardiovascular disease by complementing the information provided by traditional risk factors (4, 5, 7–9). The potential clinical implication of arterial stiffness measurements in early detection of high-risk individuals (10, 11) and in driving hypertensive patient therapy (12) make arterial stiffness measurements a promising keystone in hypertension management and cardiovascular prevention (13, 14).

Pulse wave velocity (PWV) measurement is considered the simplest, non-invasive, robust, and reproducible method for assessing arterial stiffness, with the carotid-femoral vascular bed regarded as the most easily accessible pathway for aortic PWV measurements (2, 15). Because of the abundance of population data and reference values for the healthy population (16), carotid-femoral pulse wave velocity (cfPWV) recorded with the SphygmoCor CvMS applanation tonometer has been established in the literature as a reference for comparison and is recommended by the ARTERY Society guidelines as a standard against which new PWV devices can be validated (17). Devices that estimate PWV from other vascular beds and are technically less demanding, such as those that estimate aortic PWV (PWVao) from brachial cuff-based waveform analysis recorded at a single site—e.g., oscillometric Arteriograph device, are also commonly used in practice. While both the SphygmoCor CvMS and the Arteriograph device have been verified using invasively recorded aortic PWV (18, 19), their measurements are not interchangeable (20–22), likely because they employ different measuring techniques. For this reason, head-to-head comparison between the devices is hard to interpret.

Despite its considerable potential for cardiovascular disease prevention, measurements of PWV have limited use in clinical practice. The updated 2021 ESC Guidelines on cardiovascular disease prevention in clinical practice argues against widespread use of PWV measurement in clinics because of difficulties in measurements and precision of measurements (23). While recommendations for minimizing confounding of arterial stiffness measurements in order to obtain reliable PWV values have been published and are included in study protocols (24), there are additional factors that have been proposed to alter PWV measurement (24–28). These factors, that are not controlled for in a protocol, may increase between- and within-subject variability of PWV measurements, lowering the device’s resolution to detect a minimal clinically important change and decreasing precision of PWV measurements, which consequently reduce the power of a study. As demonstrated by the SPARTE study’s low power (12), improving the power of longitudinal studies measuring PWV and reducing PWV-related measurement difficulties (e.g., the need to repeat the measurement) are critical to facilitating large longitudinal studies and improving PWV translation into clinical practice. Aside from clinically relevant PWV changes, factors such as mean blood pressure (MAP), heart rate (HR), different observers, time of day, or outdoor temperature have been implicated as additional sources of PWV variability (24, 25, 29–35). While individual factors have been studied in few studies, no study has systematically examined several factors together to assess the independent contribution of each factor while controlling for the others. To minimize the impact of additional factors that significantly affect PWV measurements in a clinical setting it is important to identify such factors and quantify their effects. Furthermore, it is essential to identify and control factors affecting measurement difficulties of a device, such as the need to repeat a PWV measurement, in order to ease the use of devices in clinical setting. Because different PWV devices, particularly those that use different measurement principles, do not necessarily have interchangeable PWV values (20–22), it is reasonable to assume that the aforementioned analyses will produce different results and should thus be performed separately for each device. The study had two goals: (a) to identify factors that affected PWV measurements that differed from those controlled in the standard PWV measurement protocol (e.g., meals, smoking, exercise), and to quantify their effects; and (b) to determine which of these factors affects a device’s measurement difficulties (e.g., the need to repeat PWV measurement, or to manually select a signal for analysis), and to what extent. The experimental setting and meteorological conditions, as well as physiological factors and participant characteristics, were systematically assessed as factors. The analyses were performed separately for the two devices that use different measuring techniques—the applanation tonometer SphygmoCor CvMS and the oscillometric device Arteriograph.

To reach our goals we used the study design that yields strong evidence: a block-randomized cross-over longitudinal study with two observers that were blinded to each other’s readings, the largest number of repeated measurements per participant (N_per participant_ = 12 recordings; N_total_ = 420), uniform distribution of participants by age, sex, BMI and hypertensive status, and multilevel mixed-effects models used in data analysis. All the recordings were performed over 2 weeks. In such a short period of time, clinically relevant changes in vascular biology are not expected, so the variability of PWV during repeated recordings can primarily be attributed to chance and confounding factors.

## 2. Materials and methods

### 2.1. Participants

**FIGURE 1:**
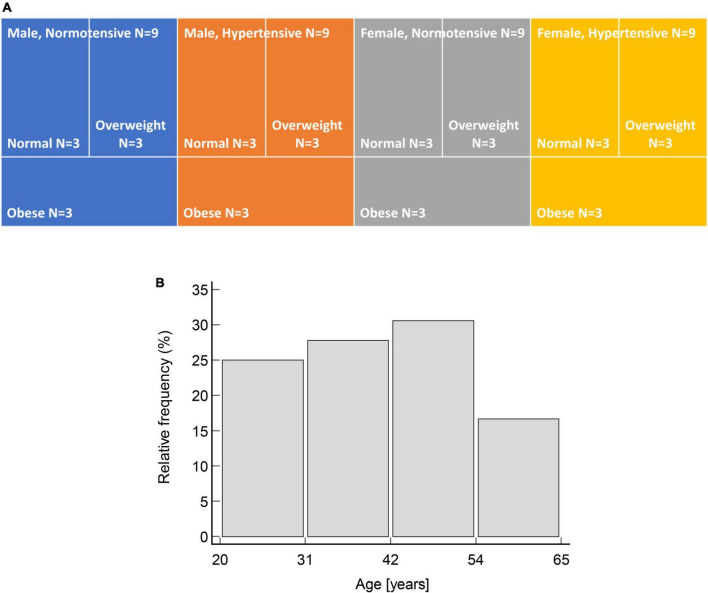
Uniform distribution of participants by: **(A)** Sex, hypertensive status and BMI categories; and **(B)** age (χ^2^ = 1.5, *p* = 0.692).

The study enrolled 36 participants aged 20–60 years. Participants were purposively sampled by age, sex, hypertension status (normotensive or hypertensive), and body mass index (BMI, ranging from normal weight to obese) to ensure even distribution across the inclusion criteria (Figure 1). Participants’ hypertension status was established by self-report of physician diagnosis, with all such persons reporting receiving treatment, whereas BMI categories were determined according to the Centers for Disease Control and Prevention classification for adults (36). Exclusion criteria included self-reported arrhythmias, cerebrovascular disease, pregnancy, surgery amputation, oncology disease, psychiatric disease, and infections throughout the trial duration.

One person (female, 45 years old, hypertensive and obese) who was initially enrolled in a study was additionally later excluded because she contracted an infection over the course of the study, leaving a total of 35 participants.

The study was approved by the Ethics Committee at the University of Split School of Medicine, and all participants provided written informed consent.

### 2.2. Study design

This is a single-blind block-randomized cross-over longitudinal study.

The study took place between October 2019 and February 2020 at the University of Split School of Medicine in the Laboratory for vascular aging.

Each participant was recorded 12 times in total over the course of 2 weeks, four times during each of the 3 visit days that were separated by 1 week. On each visiting day, recordings were taken in the morning (7–10 h) and afternoon (16–18 h) by the two observers.

The order of devices—Sphygmocor CvMs and Arteriograph and of observers, was randomized using the block size of 4. The two observers were blinded to each other’s readings.

### 2.3. PWV measurements

Pulse wave measurements were taken with the two devices that use different measuring techniques—the applanation tonometer SphygmoCor CvMS (Atcor Medical, Sydney Australia) with which we collected cfPWV data and the oscillometric device Arteriograph (TensioMed, Budapest, Hungary) used to collect PWVao data.

The measurements were taken in accordance with the American Heart Association’s recommendations for improving and standardizing vascular research on arterial stiffness (24). The observers performed measurements in a quiet, temperature-controlled room at a comfortable temperature of 21–23°C. The participants rested in the supine position for 10 min before the first PWV measurement to ensure hemodynamic stability. After completion of the series of measurements with one device, participants were asked to stand up, walk around the room, and then rest supine for 10 min to prepare for measurements with the second device. This step was necessary to prevent participants from falling asleep while resting supine for an extended period, especially in the morning. During the measurements, participants were asked not to talk or sleep. All measurements were performed on the right hand (Arteriograph) and the right carotid and femoral artery (SphygmoCor).

Participants were requested to abstain from vigorous exercise and alcohol consumption for at least 24 h prior to a recording session (morning or afternoon). They were also instructed not to eat or drink anything except water or smoke for at least 3 h before any recording session. Those taking vasoactive medicines were advised to continue taking them as usual and not to change the dosage during the study.

Before the start of the study, both observers had received extensive 7-day training during which they performed approximately 40 high-quality measurements under supervision.

To calibrate the pulse wave signals acquired by the SphygmoCor, we obtained brachial blood pressure measurements with the validated oscillometric sphygmomanometer (Welch Allyn Connex ProBP 3400 digital blood pressure monitor with SureBP technology).

**FIGURE 2:**
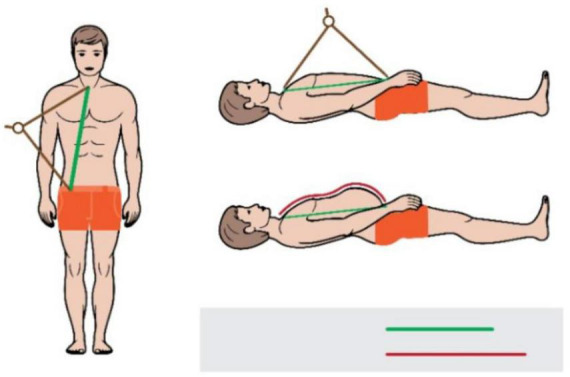
Measurement of a distance between the sternal notch and the femoral site with a tape (red line) and a school divider (green line).

We used the subtracted distance method to calculate wave travel distance. The method was chosen over the direct method as per recommendation by the latest guideline (24). A large school divider (Figure 2) was used to measure the distance between the sternal notch and the femoral measurement site, as well as the distance between carotid measurement site and the sternal notch was than subtracted from this distance. The tool was selected because it measures straight-line distance independent of body shapes such as particularly large bellies and/or breasts in obese individuals. It is similar to a sliding caliper, which has been recommended for use when straight-line measurement with a tape measure is not possible (24, 37, 38). However, unlike slide caliper whose slide blades may still impede with the body shape in overweight and obese individuals to some extent, a divider is unaffected by it due to its long arms. We only measured the distance between the carotid and femoral sites during the first visit. Each observer obtained a single distance measurement during this visit. If the measured distances differed between observers, the process was repeated, and if the difference remained after the second round of measurements, the average of the four previous distance measurements was used as the true distance (*N* = 1 participant).

### 2.4. Collection of other data

All participants underwent a medical history.

**FIGURE 3:**
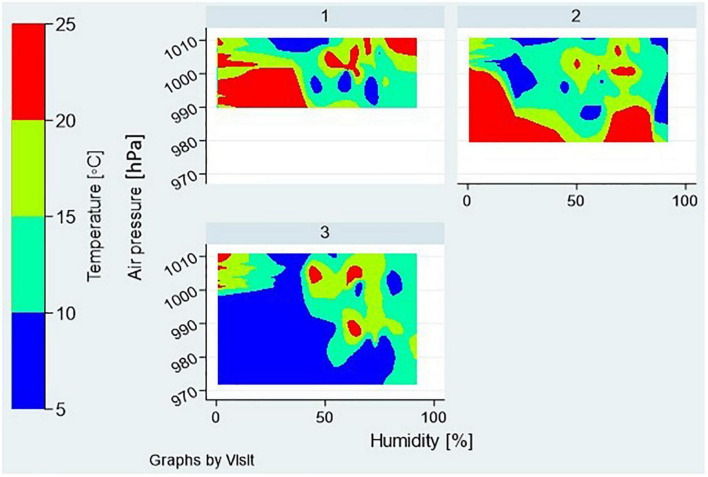
Meteorological factors during the 3 visit days—distributions of outdoor temperature, air pressure, and relative humidity are shown as contour plots for all the participants’ visits assigned to a particular visit day—1, 2, or 3.

The meteorological data: outdoor temperature, air pressure, and relative humidity, were provided by the Meteorological and Hydrological Service of Croatia’s local office, and used to estimate weather conditions on each visit day and time. The contour plot is used to show the distributions of three meteorological parameters by a visit day (visit 1, 2, or 3) for all of the participants’ visits, with relative humidity and air pressure as x and y dimensions, and temperature as a color coded z-dimension (Figure 3). The graph shows that outdoor conditions on the visit day 3 differed significantly from those on the visit days 1 and 2 (Figure 3).

### 2.5. Sample size consideration

The study employs a multilevel data structure, including 35 groups (participants, level-2) and 12 repeated measurements by a participant (level-1) totaling 420 observations. Because no random slopes are anticipated in any of the multilevel models that we developed, the sample sizes mentioned above (levels 2 and 1) are deemed adequate for estimating unbiased and accurate regression coefficients, variance components, and first level standard errors (39, 40), allowing for models with up to 12 independent variables (41).

### 2.6. Data analysis

We used descriptive statistics to describe distribution of quantitative (mean and standard deviation or median and IQR, depending the shape of distribution) and qualitative (absolute and relative frequencies) variables. To identify predictors of PWV single readings, or different types of measurement difficulties we employed several multilevel models with random intercept. Depending on the type of a dependent variable we used multilevel mixed-effects generalized linear models for continuous dependent variables like PWV readings, mixed-effects logistic regression models for dichotomous dependent variables like the occurrence of marginal-quality signals or manual signal selection, and mixed-effect Poisson regression for count data like the count of repeated attempts to record PWV values due to low-quality of a captured signal. All of the models were run with the robust estimator, which is resistant to certain types of misspecification in multilevel models such as heteroscedasticity or deviation from normality (42, 43), and sensitivity analysis was performed with the maximum likelihood (ML) method without robust estimator.

Each model was built in two steps. The experimental setting variables—order of visit, time of day, first device used in a session, and observer, meteorological variables—outdoor temperature (^°^C), air pressure (Pa), and relative humidity (%), and participants’ characteristics—age, sex, BMI, hypertension status, MAP, SBP, DBP, HR—were all investigated for their relationship to a dependent variable by a simple multilevel regression analysis. Those independent variables that were associated with dependent variable at the *p* < 0.2 significance level, entered into multiple multilevel regression model. For independent variables that were non-significant in a multiple model, their contribution to the model (pseudo R^2^) was investigated further to decide on their inclusion in the final model.

The metrics for assessment of individual variability of PWV readings was determined by considering the multilayered data structure. The average within-subject CV was calculated using the root mean square method (44). The intraclass correlation coefficient (ICC) was calculated by using random-effects model to estimate correlations between average measurements made on the same participant. As outliers may significantly affect this measure, we excluded the severe outliers from the calculation (45).

## 3. Results

The study included a total of 35 participants. Recorded PWV values ranged from 4.5 to 10.8 m/s for cfPWV and 5.5–15.8 m/s for PWVao. The validation sample also covered wide ranges of brachial blood pressures, age and BMI that were uniformly distributed (Figure 1). Participants’ characteristics are shown in Table 1.

**TABLE 1:** Characteristics of participants, *N* = 35.

Characteristics	Md (range) or N (%)
Age (years)	41 (20–60)
**Sex**	
Females	17 (49%)
Males	18 (51%)
BMI	27.3 (19.4–38.9)
**Hypertension status**	
Hypertensive	17 (49%)
Normotensive	18 (51%)
bSBP (mmHg)	126 (98–177)
bSDP (mmHg)	72 (53–98)
HR (beats per minute)	67 (48–94)

BMI, body mass index; bSBP, brachial systolic blood pressure; bDBP, brachial diastolic blood pressure; HR, heart rate.

The difference in distance between the carotid and femoral sites measured with a tape vs. a school divider strongly correlated with the level of obesity as evaluated by BMI (*r* = 0.79, *P* = 0.020). Thus, the school divider was effective in removing this effect of BMI.

Overall, the coefficient of variation for within-subject variability was 9.9% (95% CI 9–11%) and 5.3% (95% CI 5–6%) for cfPWV and PWVao measurements, respectively; while ICC for within-subject average cfPWV measurements was 94% (95% CI 91–97%) and 96% (95% CI 94–98%) for PWVao measurements.

### 3.1. Factors significantly affecting PWV measurements

Table 2 lists the factors with a significant effect on PWV measurements, that were identified among experimental conditions, physiological and meteorological factors, as well as characteristics of the participants that we assessed in this study.

**TABLE 2:** Factors that significantly affect PWV measurements, and expected between- and within-subject differences in PWV measurements that are due to a factor’s observed range in a sample and its within-subject variability.

Device	Factor	B	95% CI for B	*P*-value		Between-subjects difference in PWV due to a factor, calculated from predicted margins^†^ (m/s)	Within-subject average difference in repeated measurements due to within-subject variability of a factor (m/s)
SphygmoCor cfPWV (m/s)	Age (year)	0.04	0.02	0.06	<0.001*	1.63	No variability of the factor, but increase in variability of repeated cfPWV measurements with age
	MAP (mmHg)	0.05	0.02	0.08	<0.001*	1.14	In interaction
	Hypertension (yes vs. no)	0.44	-0.02	0.91	0.062**	0.44	No variability
	Outdoor temperature (^°^C)	0.18	0.06	0.31	0.005*	-0.25	In interaction
	Order of visit						
	2nd vs. 1st	-0.23	-0.43	-0.03	0.023*	−0.23	−0.03 (−0.25, 0.20) ^††^
	3rd vs. 1st	-0.05	-0.21	0.11	0.555	–	–
	Interaction Outdoor temperature × MAP	-0.002	-0.004	-0.001	0.003*	2.62 m/s at 5^°^C and 0.04 m/s at 25^°^C^§^	At 5^°^C average difference in repeated measurements due to variability in MAP is 0.27 m/s (0.24–0.30), and at 25^°^C 0.004 (0.004–0.005) m/s
	Snijders/Bosker R^2^ Level 1: 39%, Level 2: 59%						
Arteriograph PWVao (m/s)	Age (year)	0.08	0.06	0.10	<0.001*	3.15	No variability of the factor, but increase in variability of repeated PWVao measurements with age
	MAP (mmHg)	0.04	0.02	0.05	<0.001*	2.30	0.23 (0.10–0.33)
	Height (cm)	0.06	0.02	0.09	0.001*	2.14	No variability
	Sex (female vs. male)	1.56	0.69	2.42	<0.001*	1.56	No variability
	HR (bpm)	0.02	0.002	0.04	0.031*	1.45	0.12 (0.01–0.27)
	Snijders/Bosker R^2^ Level 1: 50%, Level 2: 67%						

B, regression coefficient. ^†^Difference in predictive margins of PWV for an observed range of a factor, adjusted for other factors. ^††^Values in the brackets are calculated considering uncertainty of estimates for regression coefficient and factors’ within-subject variability. ^§^For the interaction term we calculated the maximal expected changes in cfPWV given the MAP range: at 5 and 25^°^C; Significant at the: *0.05, **0.1 level.

**FIGURE 4:**
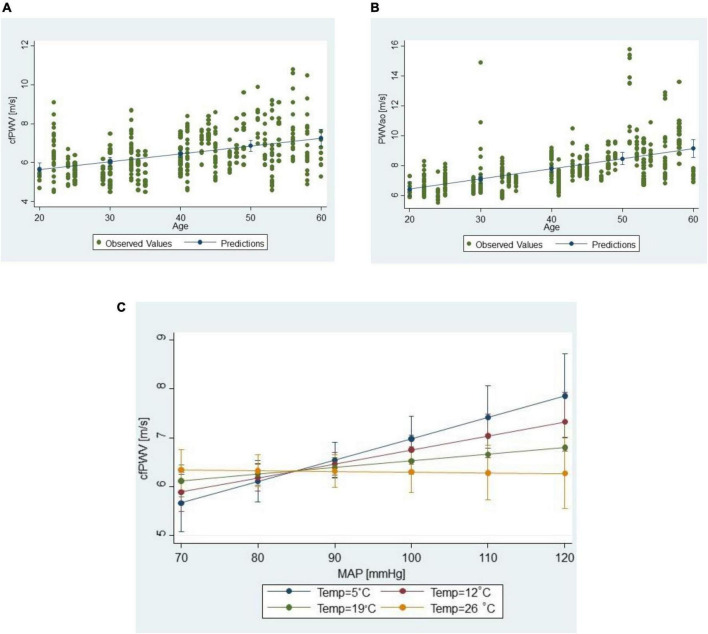
Scatter plots of **(A)** cfPWV measurements vs. age, and **(B)** PWVao measurements vs. age; **(C)** moderation interaction of the outdoor temperature on the relationship between cfPWV values and MAP; lines connect predictive margins of the multilevel regression model with 95% CI.

In both the cfPWV and PWVao models, the most prominent effects to which the largest difference in predicted PWV margins was attributed were age and MAP, in that order. The increase in PWV values with a 1-year increase in age was twice higher for PWVao than cfPWV measurements (Table 2, see 95% CI for B; Figures 4A, B). However, we could not directly compare the effects for MAP as the factor was involved in a significant interaction with outdoor temperature in the cfPWV model, which hampered interpretation of its main effect (Figure 4C).

As for the meteorological factors, only the outdoor temperature had a significant effect on cfPWV measurements. In cfPWV model, as mentioned earlier, outdoor temperature was involved in the interaction with MAP, suggesting that it modifies the relationship between cfPWV and MAP (Figure 4C). PWVao measurements were not influenced by any outdoor meteorological factor.

Except for the order of visit in the cfPWV model—notably the comparison of 2nd to 1st visit values, no other experimental factor was associated with differences in cfPWV or PWVao measurements.

Increased values of PWVao measurements were also associated with increased values of BMI and HR, and a female sex, whereas in the cfPWV model it was hypertensive status that was found to significantly increase cfPWV.

During the development of multilevel mixed-effects models we also assessed if there was a significant interaction between sex and age. After including an interaction term between sex and age in the full multivariate models of both SphygmoCor and Arteriograph, and controlling for menopause status, we discovered that the interaction between age and sex is not significant (*p* ≥ 0.365).

Finally, after developing multilevel mixed-effects models that aimed to identify factors influencing values of PWV measurements, we also evaluated which, if any, participants’ characteristics are related to variability of repeated measurements in a person. We found for both cfPWV (Pearson’s *r* = 0.51, *p* = 0.002) and PWVao (*r* = 0.34, *p* = 0.047) measurements that older age was associated with wider range of repeated PWV measurements observed in a person, whereas BMI, hypertension status or sex were not associated with this variability. However, we did find significantly larger variability of within-subject PWV measurements in menopausal than in women with menstrual cycle, for both devices (*P* ≤ 0.035, Mann-Whitney test). Moreover, for women who menstruate, within-subject PWV variability was comparable to men (*P* ≥ 0.310). In addition, we also assessed if the order of visit, the only experimental condition significantly affecting cfPWV values, is associated with decreasing variability of cfPWV measurement in a person and found this was not the case (repeated measurements ANOVA, *P* = 0.781).

Regarding the repeated measurements defined by the final models depicted in Table 2, within-subject cfPWV on average deviated by 0.73 m/s (95% CI 0.64–0.83), whereas PWVao deviated by 0.84 m/s (95% CI 0.58–1.20). These models described 59 and 67% of the repeated variability, respectively.

### 3.2. Factors significantly affecting difficulties in measuring PWV

Next, we wanted to know which factors influenced difficulties in measuring PWV with each device. We focused on the following difficulties: (a) the number of repeated measurements as a result of the SphygmoCor’s pulse wave velocity signal failing the quality control, (b) the possibility of observing the marginal quality signal by SphygmoCor with the coefficient of variation for cfPWV estimation between 6 and 10%, or (c) the Arteriograph’s oscillometric signal being manually selected for analysis (Table 3).

**TABLE 3:** Predictors of the measurement difficulties for Arteriograph and SphygmoCor devices.

Device	Predictors	OR	95% CI		*P*-value
Arteriograph—a need to manually select signal for analysis	Sex (female vs. male)	51.44	6.42	412.21	<0.001*
	Time of the day (afternoon vs. morning)	0.23	0.10	0.54	0.001*
	Measurer (no. 2 vs. no. 1)	1.91	1.23	2.95	0.004*
	Mixed-effects logistic regression model				
		IRR	95% CI		*P*-value
SphygmoCor—a need to repeat a measurement	Sex (female vs. male)	1.23	1.01	1.50	0.040*
	Time of the day (afternoon vs. morning)	0.88	0.79	0.97	0.014*
	Order of visit				
	2nd vs. 1st	0.95	0.86	1.06	0.361
	3rd vs. 1st	0.86	0.77	0.96	0.006*
	Mixed-effects Poisson regression model				
		OR	95% CI		*P*-value
SphygmoCor—occurrence of a marginal signal quality with cfPWV CV between 6 and 10%	Time of the day (afternoon vs. morning)	0.60	0.35	1.03	0.065**
	Order of visit				
	2nd vs. 1st	1.00	0.51	1.95	>0.999
	3rd vs. 1st	0.50	0.26	0.99	0.045*
	Mixed-effects logistic regression model				

CV, coefficient of variation; OR, odds ratio; IRR, incidence rate ratio.

Significant at the: *0.05, **0.1 level.

As opposed to the automatic selection of the oscillometric pulse wave signal by Arteriograph device, the manual signal selection is time-consuming and subject to uncertainty. In total, we had to manually select a signal for analysis in 78 (19%) of the Arteriograph recordings. We did not find, however, that manual selection affected PWVao values when this factor was added as independent variable to the simple (*p* = 0.438), or final (*p* = 0.276) multilevel model. The results demonstrate that it is very likely that the Arteriographs’s oscillometric signal will be manually selected for analysis in a female participant. In fact, manual adjustment was required at least once for 11 (65%) of the women, with five women requiring it on ≥8 instances out of 12 measurements. In contrast, just five males (28%) required this modification on 1–3 occasions. Measuring in the afternoons was strongly to moderately associated with a lower chance of manual adjustment, reducing the odds ratio for it by 77% (95% CI from 46 to 90%). Finally, a chance for manual adjustment differed significantly between the measurers by increasing OR for the measurer 2 by 0.91 (95% CI 0.23–1.95).

In terms of the SphygmoCor, with which we were able to record all 12 measurements for each participant, we had to repeat a measurement 135 (32%) of the time. Female sex increased the incidence rate ratio (IRR) of a repeated measurement by 23% (95% CI 1–50%), whereas afternoon recording time and 3rd vs. 1st visit decreased IRR by 12% (95% CI 3–21%) and 14% (95% CI 4–27%), respectively.

We also discovered that the same two factors—afternoon recording time and 3rd vs. 1st visit—decreased the odds of a signal with marginal quality, which we observed in 95 (23%) of measurements, by: 40% (OR 95% CI −3 to 65%) and 50% (95% CI 1–74%), respectively. When the marginal quality of a signal was added as independent variable to the simple and final multilevel model we found that it increased cfPWV values by on average 0.37 m/s (95% CI 0.16–0.58, *p* = 0.001).

## 4. Discussion

We systematically examined a number of experimental and meteorological conditions as well as physiological factors and participants’ characteristics to find if, and to what level, they affect (a) recorded PWV values, and (b) measurement difficulties associated with this recording. PWV values were repeatedly collected from the enrolled participants over the course of 2 weeks during which no clinically relevant change in PWV values is expected. The analyses were done separately for measurements acquired with two validated devices: cfPWV measurements acquired with applanation tonometry device SphygmoCor CvMS and PWVao values recorded with oscillometry Arteriograph device.

We utilized the study design that provides the strong evidence: block-randomized, cross-over, longitudinal study with as many as 12 repeated measurements per participant per device, recorded with observers blinded to each other’s readings. Furthermore, our validation sample exhibited a reasonably wide range of PWV, age, BMI and brachial blood pressures values, and uniform distributions across age, sex, BMI, and hypertension status. Given that uniform distribution puts less emphasis on the center of the distribution and more on its extremes, it produces more precise validation estimates and is thus preferable as validation sample to a sample representative of an underlying population (46).

There haven’t been many studies that look at the short-term repeatability or reproducibility of PWV measurements (47–51), with studies reporting from 2 to 6 repeated measurements per participant. Despite the fact that we recorded the most repeated PWV measurements per participant (*N* = 12), and that our sample had a reasonably wide range of PWV and PWV determinants, which tend to increase observed variability of PWV measurements, the agreement of repeated PWV measurements estimated in our study was comparable to what has been reported in the literature. Grillo et al. estimated within-subject CV, and ICC for cfPWV values recorded with SphygmoCor CvMS device in patients of predominantly normal weight who were hospitalized for suspected coronary artery disease. They used six repeated measurements per person and reported comparable metrics to our study with the CV of 9.5 (95% CI 7.7–11.0), and ICC of 0.85 (95% CI 0.78–0.90) (48). Our ICC value for the cfPWV measurements was also comparable to that reported by studies that were performed on patients with peripheral arterial disease (52, 53). As for the Arteriograph’s PWVao measurements, Li et al. measured PWVao at 3 timepoints during a day in 70 participants including healthy young and elderly participants, and patients with cardiovascular disease treated in outpatient clinic; and reported CV of 6.1%, which is comparable to our study (54). Similarly, Ring et al. reported CV of 9.3 and 9.6% in 51 healthy non-smoking participants for Arteriograph’s PWVao and SphygmoCor’s cfPWV, respectively (55).

### 4.1. Factors affecting PWV readings

#### 4.1.1. Age

Age was the factor with the largest effect on cfPWV and PWVao measurements, followed by MAP. This is consistent with the data from other studies that show a strong dependence of PWV on age and MAP (16, 24, 56, 57). On average, age accounted for approximately 2–3 m/s PWV difference between subjects, with apparently stronger effect of age on PWVao values: cfPWV increased by 0.4 m/s (95% CI 0.2–0.6), and PWVao by 0.8 m/s (95% CI by 0.6–1.0) every 10 years.

Other studies utilizing the SphygmoCor CvMS device reported comparable effects of age on cfPWV measurements ranging from: 0.2 m/s per 10 years (58), to 0.3–0.4 m/s (59), to somewhat larger effects of around 0.7–0.9 m/s reported by the same group of authors (16, 60). While age was identified as the strongest determinant of PWVao too (61), studies that investigated factors influencing Arteriograph’s PWVao measurements did not reported comparable, unstandardized regression metrics. However, the study that investigated PWVao measurements recorded with oscillometric Vicoder device reported a comparable effect of 0.4–1.0 m/s per 10 years (62).

While we may assume that age remained constant during the 2 weeks of the study and thus did not contribute to an absolute change in a person’s repeated measurements, we also discovered that older age increased the variability of repeated measurements for both cfPWV and PWVao, thereby increasing measurement error. Grillo et al. discovered that patients with increased arterial stiffness had greater variability in repeated PWV measurements acquired with various cfPWV-estimating devices (48). This relationship is thought to be due to the fact that PWV is defined as a ratio of traveled distance to pulse wave transit time (PWTT), in which case a small difference in PWTT can cause a relatively large difference in PWV in subjects with high arterial stiffness, whereas this difference is negligible in subjects with normal arterial stiffness. As age is the strongest determinant of PWV, the association reported by Grillo et al. corroborates the association between age and within-subject short-term PWV variability that was reported in our study. Because both of the devices we tested estimate PWTT: SphygmoCor by determining pulse transit time from carotid to femoral location, and Arteriograph by determining the time difference between the first systolic wave and the second reflected wave; we found the said association for both devices. This finding suggests that when patients are monitored for longitudinal changes in PWV, it would be advantageous to increase the number of measurements in older persons during one visit (from 2 to 3–4) to improve precision of estimated PWV.

#### 4.1.2. MAP and outdoor meteorological factors

The second strongest effect on PWV recording was due to MAP, which is considered the most significant physiological variable affecting arterial stiffness (30, 31, 34). With the increase of MAP, vessels stiffen, meaning that the effect of this variable on repeated PWV measurements should be considered whenever the measurements are taken under different BPs. MAP on average accounted for approximately 1–2 m/s PWV difference between subjects in our study, with PWVao increasing by 0.2 m/s (95% CI 0.10–0.25) for every 5 mmHg. This is comparable to the effect estimated with Vicorder, another oscillometric device: 0.05–0.20 m/s per 5 mmHg (62). Considering within-subject variability of MAP in our study, on average 0.23 m/s (from 0.10 to 0.33 when uncertainty in estimates is considered) discrepancy in repeated PWVao measurements may be assigned to MAP variations.

The expected changes in cfPWV due to MAP or outdoor temperature changes were less obvious due to the significant interaction between outdoor temperature and MAP, demonstrating that outdoor temperature moderates the relationship between cfPWV and MAP. The expected difference between predicted cfPWV assigned to lowest and highest observed MAP and adjusted for other factors at 5^°^C was 2.62 m/s, but at 25^°^C this difference was only 0.04 m/s. Individual differences in repeated measurements that are due to MAP variations are predicted to be 0.27 m/s (uncertainty 0.24–0.30) at 5^°^C and 0.004 m/s (0.004–0.005) m/s at 25^°^C. Interestingly, there was no significant main effect of outside temperature or its interaction with MAP for PWVao measurements.

While the negative relationship between outdoor temperature and BP readings has been observed in many studies (63–67), similar relationship was hypothesized for arterial stiffness, but studies reported inconsistent results. Di Pilla et al. reported that in an unadjusted regression analysis, cfPWV recorded by SphygmoCor was weakly and inversely associated with outdoor temperature, but not in a multiple regression analysis that controlled for age, BMI, SBP, DBP, daylight hours, O_3_, CO, and N_2_O (25). In repeated measures ANOVA analysis, Kita et al. demonstrated considerable sessional change of an arterial stiffness index—arterial velocity pulse index, with higher stiffness observed during a summer (26). The omission of a significant interaction term between temperature and MAP in these models may explain inconsistencies, as exclusion of the term from cfPWV model in our study also resulted in a non-significant main effect of temperature, while a simple correlation also showed that cfPWV is inversely associated with temperature. While the mechanism underlying this relationship is beyond the scope of our study, it has been proposed that cold-induced sympathetic activation may account for the dependence of cfPWV readings on outdoor temperature in conditions where the room temperature is constant (25, 68). Having said that, it appears that at lower outdoor temperatures, resting time in supine position in the temperature-controlled room might be extended beyond 10 min to allow for temperature accommodation, or alternatively, repeated measurements might be taken in a season with higher outdoor temperatures, such as those around 25^°^C. More research with different adaptation times is needed to determine whether temperature accommodation is indeed responsible for the observed effect.

#### 4.1.3. HR

Aside from the effect of outdoor temperature on PWV measurements, there was also a disparity in the effect of HR between the cfPWV and PWVao models. This physiological factor had a moderate effect on PWVao readings explaining on average 1.45 m/s difference in PWVao between the subjects with PWVao increasing on average by 0.2 m/s (95% CI 0.02–0.40) per 10 bpm. The effect is similar to that reported by Tan et al., who used a hybrid applanation tonometry/oscillometric device SphygmoCor XCEL device and found an increase of cfPWV of 0.11–0.28 m/s per 10 bpm (35). In addition, individual variability in HR in our study accounted on average for 0.12 m/s (uncertainty, 0.01–0.27) discrepancy in repeated PWVao measurements. Considering these effects, PWVao is expected to have minimal physiologically relevant changes for small changes in HR, while larger changes in HR may be viewed as leading to considerable differences in PWVao. However, we did not observe a significant effect on cfPWV readings.

While current PWV estimation guidelines recommending that HR be considered as a confounding factor, there is still disagreement about the effect of HR on aortic PWV measurements, particularly considering that short-term studies investigating the relationship between heart rate and arterial stiffness reported varying results, including a positive, negative, and no association (24). O’Rurke et al. proposed that the apparent association between aortic PWV and HR might be due to systematic error introduced by certain devices when estimating such PWV (32), whereas Salvi et al. demonstrated, on data collected with applanation tonometry device PulsePen, that the said association was cofounded by the ventricular ejection time (69). Tan et al., however, used hybrid SphygmoCor XCEL and demonstrated BP-independent effects of HR on cfPWV (35). In line with the O’Rurke’s considerations, our results on two different devices suggest that the usage of devices with differing PWV measurement techniques might be the source of this disparity in findings. While many studies employing SphygmoCor CvMS or applanation tonometry technique in general reported no significant change in cfPWV with HR (60, 69, 70), studies that estimated aortic PWV using Doppler method (71), Arteriograph’s oscillometric technique (61) or a hybrid applanation tonometry and oscillometric technique such as the one employed by SphygmoCor XCEL device (35) found independent HR effect on PWVao.

#### 4.1.4. Experimental conditions

Out of all the experimental conditions tested only the visit order affected the PWV measurements. Specifically, whereas the order of visits had no effect on the PWVao measurements, the cfPWV measurements acquired during the second visit were on average 0.23 m/s (95% CI 0.03–0.43) lower than those of the first visit. However, no significant difference was found when the cfPWV data of the third visit were compared with those of the first visit. One possible explanation for the lack of a trend on the third visit is a confounding of this effect by outdoor temperature. Because outdoor temperature was identified as an important factor influencing cfPWV measurements, more pronounced meteorological changes on the third visit likely masked the effect of a visit order. In particular, for some subjects, the outdoor temperature varied by 8–10°C between the third and the other two visits.

The discovery of lower cfPWV values during the second visit may suggests that, despite receiving the amount of training recommended by current guidelines (24), both observers may have lacked some expertise in using SphygmoCor CvMS during the first visit. However, we did not observe a decrease in the within-subject variability of cfPWV measurements with increasing order of visits, as would be expected if the effect of poor training was present. Furthermore, Grillo et al. have shown that the 2-week training period is sufficient to achieve acceptable to excellent agreement of PWV recordings for various devices including SphygmoCor CvMS (48) and, as previously discussed, we have shown that the agreement of repeated cfPWV measurements estimated in our study was comparable to that reported by Grillo et al. Elliot et al. studied the influence of training on the repeatability of cfPWV values as well, but the authors assessed a hybrid SphygmoCor XCEL device with different mode of operation for which training differs (28).

Alternatively, the higher values of cfPWV during the first visit could point toward the white coat effect on arterial stiffness (72, 73). Indeed, the first measurement in our study was considerably higher than the second during the first visit cfPWV measurement series (median difference 0.35 m/s, 95% CI 0.05–0.65), and was also higher than the first measurement of the second visit (0.60 m/s, 95% CI 0.2–1.0). However, Barochiner et al. in an unadjusted analysis comparing isolated office uncontrolled hypertensive participants with sustained normotensive estimated much larger white coat effect on cfPWV with a median difference of 1.2 m/s (73). When we looked at SBP, we also found significant differences between the said measurements (*p* ≤ 0.015; 1st measurement in first visit was on average higher for 3.3 mmHg, 95% CI 0.6–6.0 than 2nd, and for 3.8 95% CI 1.0–6.5 than 1st measurement in second visit), but these differences are not large enough to be classified as white coat effect. We also found no difference in DBP between these measurements (*p* ≥ 0.422). A clinically significant white-coat effect is defined in terms of BPs as an office or clinic blood pressure exceeding the daytime ABPM by 20 mm Hg systolic or 10 mm Hg diastolic, either in the absence or presence of antihypertensive drug treatment (74). Thus, while the whitecoat effect, which describes a transient or persistent alerting reaction observed in the majority of patients, was likely present in our sample, the magnitude of it was insufficient to justify the first measurement discartion.

We did not find that time of day or different observers (conditional on observers having the same volume of training) affected cfPWV or PWVao measurements although the current guideline on PWV estimation recommends that repeated/follow-up measurements should be taken at the same time of day, preferably with the same observer (24). Several studies performed on different populations: young healthy volunteers (29), women with uncomplicated pregnancy (33), or healthy individuals of different ages and patients with heart disease (54) corroborate our result on lack of circadian variation in PWV measurements, whereas the study that reported increase in cfPWV with time of day revealed that diurnal PWV changes lost significance after adjustment for BPs suggesting that changes in arterial stiffening are mediated through changes in BP (75). As a result of the findings, future study protocols for follow-up PWV measurements could be simplified, as it is not necessary to measure cfPWV at the same time of day or with the same observer, provided that the quantity of training is adequate and comparable.

#### 4.1.5. Participant characteristics: Sex, height, and hypertensive status

The differences in the PWV models of the two devices included participant characteristics such as sex and height which had a significant effect on PWVao, but not on cfPWV measurements; and hypertensive status, which on average increased cfPWV in hypertensive participants by 0.44 m/s but did fect PWVao measurements.

Female sex on average increased PWVao by 1.56 m/s, whereas height explained on average 2.14 m/s difference in PWVao measurements between the subjects, with one cm of height increasing PWVao by 0.06 m/s (95% CI 0.02–0.09).

Sex, with higher values of PWVao in women compared to men, was also identified as significant factor in another study investigating Arteriographs’ PWVao measurements (61). However, while reports on the relationship between sex and central PWV measurements that were taken by devices other than Arteriograph were varied, all studies including those utilizing oscillometric devices reported either greater values of central PWV in men (16, 59, 60, 62, 76–78) or no association with sex (69, 79–82). Thus, it appears that higher values of PWVao measurements in women may be specific to Arteriograph device. Contrary to PWVao measurements, we did not find that cfPWV measurements were associated with sex. Vermeersch et al. estimated peripheral and central PWV with applanation tonometry in a large healthy, middle-aged population and concluded that while peripheral, carotid PWV measurements were associated with sex this was not the case with central arterial stiffness parameters such as cfPWV (80). Also, Reference Values for Arterial Stiffness’ Collaboration in 2010 did not find significant differences between sexes in cfPWV while controlling for an age and MAP (57). Although cfPWV was markedly higher in males, the presence of male gender was also accompanied by marked differences in age and BP. After correction for age and MAP, the authors found negligible influence of gender on cfPWV (0.1 m/s difference, *P* = 0.04) and proceeded with the definition of the reference value population by including all untreated participants, regardless of sex. Similarly, sex was not identified as significant predictor of cfPWV in the multivariable linear regression models controlling for age, MAP, HR in the study by Mitchell et al. (83) which is based on apparently healthy Framingham Heart Study offspring cohort (83). Focusing solely on SphygmoCor’s cfPWV measurements, the results are contradictory, with more studies reporting that in multivariate linear regression models, cfPWV increases with age similarly for both sexes (31, 80–82), others revealed that men have higher cfPWV values ranging from 0.27 to 0.72 m/s (16, 59, 60). We discovered no significant interaction between sex and age for either device.

As for the effect of height, similar to Jatoi et al. who also investigated Arteriograph’s PWVao measurements, we found an inverse correlation between PWVao and height (data not shown). However, while in our final multiple model PWVao increased with the increase in height, in Jatoi’s multiple model the association did not reach significance (61). Furthermore, our estimate of the regression coefficient of height was positive; similar to the estimates from the simple regression model developed by Mellin et al. on Vircorder measurements (62) and multiple regression model developed on PulsePen measurements in children (84).

#### 4.1.6. Overall about PWV models

Overall, the findings suggest that SphygmoCor’s cfPWV values are more sensitive to the state of the arterial tree because, in addition to age and MAP, they are also dependent on hypertensive status, the interaction between MAP and outdoor temperature, outdoor temperature, and visit order; whereas Arteriograph’s PWVao values are more dependent on individual characteristics such as sex, height, and age as they are more heavily dependent on age than cfPWV.

It should also be noted that our findings are device-specific and cannot be generalized to other seemingly similar techniques, such as the oscillometric Mobil-O-Graph, which calculates PWV using a formula, because the factors affecting within-subject PWV variability would obviously differ.

### 4.2. Factors affecting measurement difficulties

The measurement difficulties appeared relatively frequently: from 19% of cases in which Arteriograph’s oscillometric signal had to be manually selected for analysis, to over 24% of cases in which SphygmoCor’s pulse wave signal was of marginal but acceptable quality (with a coefficient of variation for cfPWV estimates ranging from 6 to 10%), to 32% of cases in which SphygmoCor’s recordings had to be repeated. While all of these difficulties are time-consuming, we also found that one of them—a marginal quality of SphygmoCor’s signal, affected accuracy of estimated cfPWV values by raising cfPWV values on average by 0.37 m/s (95% CI 0.16–0.58 m/s). Concerning the difficulty in manually selecting the Arteriograph’s signal, which increases the uncertainty of PWVao estimation and could be expected to affect the accuracy of PWVao readings, we did not find that it was associated to a change in PWVao levels.

All of aforementioned measurement difficulties were reduced when PWV measurements were performed in the afternoon (16–18 h) compared to the morning (7–10 h) sessions. The effect of time of day on need to manually select Arteriograph’s signal was strong with afternoon session reducing the OR for automatic selection by on average 75%, and reducing the probability of the event from 23% during morning sessions to 6% in the afternoon. The time of day also had moderate effect on a need to repeat a SphygmoCor measurement by reducing its IRR in the afternoon measurements on average by 12%. Finally, the OR for the marginal quality of SphygmoCor’s signal reduced by 40% in the afternoon, with a probability of such a signal decreasing from 26% during mornings to 19% in the afternoon. As time of day did not show significant effect on absolute PWV values of either cfPWV or PWVao, just by performing measurement in the afternoon measurement difficulties could be significantly reduced.

Sex was another factor that affected measurement difficulties for both devices. It strongly increased the OR of manual selection of a Arteriograph’s signal by 51 times, and also increased IRR of repeated measurements by SphygmoCor by 1.23 times. The difficulties we observed in obtaining PWV estimation for female participants with both devices might be attributed to men being more likely prototype examinees due to the menstrual cycle’s effect on PWV recordings in women, which, as a result, would make signal acquisition and processing suboptimal for women. However, because the literature describes a validation sample rather than a developmental sample for prototypes, and the manufacturers do not provide a description of developmental sample, there is a lack of evidence to support or refute this assumption.

We also found that the order of visits, which affected repeated cfPWV measurements, affected the occurrence of measurement difficulties with the SphygmoCor too, in such a way that both the need for repeated measurement and the occurrence of a signal with marginal quality were significantly reduced during the third visit compared with the first visit, whereas the frequency during the second visit was comparable to that during the first visit. So it seems that in terms of measurement difficulties with SphygmoCor, there is still a learning curve to be observed in the first 2 weeks.

Finally, while inter-observer differences in PWV readings are not expected for oscillometric devices nor were observed in our study, we show that the need for manual selection of the Arteriograph’s signal is observer-dependent with one observer increasing the odds of it by 91%. Whether this difference between observer is due to positioning or adjusting the cuff should be explored in further studies.

Considerations about the supine and sitting positions with Arteriograph.

While we did not examine the effect of supine and sitting positions on Arteriograph’s PWVao measurements, Arteriograph is frequently used in clinic in a sitting position. Nurnberger et al. estimated the mean difference in PWVao between supine and sitting positions to be −0.18, with the limits of agreement ranging from −1.55 to 1.21 (85). Such wide limits include differences of more than 1 m/s thus demonstrating that PWVao values recorded in two different positions are not the same and only one position, preferably supine, should be used when assessing PWVao.

### 4.3. Limitations

Potential limitation of our study is referred to the pre-training of observers. Despite providing observers with the necessary pre-training, we discovered a learning curve with the SphygmoCor device, as measurement difficulties were significantly reduced during the third visit compared to the first. Furthermore, we also discovered that repeated cfPWV measurements recorded during second visit were on average 0.23 m/s lower than those recorded during the first visit, indicating that there may be a learning curve affecting cfPWV accuracy too, but we were unable to show this tendency for measurements collected during the third visit compared to first. Nonetheless, even if this learning curve affected repeated measurement accuracy, we do not expect it to have a significant effect on our results: the estimated difference in cfPWV between second and first visit is not large, we did not find that variability of repeated measurements taken during one visit was related to visit order, and we reported comparable agreement between repeated measurements to a study that reported this agreement using observers for which it proved they received a sufficient training period (48). In addition, estimated decrease in within-subject cfPWV measurements during 2nd vs. 1st visit was just −0.03 m/s (uncertainty −0.25, 0.20).

To capture short-term variability of PWV, we monitored within-subject PWV changes for 2 weeks only. Consequently, we were not able to control an effect of menstrual cycle on arterial stiffness by studying all women who still have menstrual periods at a similar phase in the menstrual cycle as this would imply that repeated measurements are also taken at similar phase of the cycle, which would prolong the monitoring period. However, we do not expect that effect of menstrual cycle substantially affected PWV variability in young women as we found significantly larger variability of within-subject PWV measurements in menopausal women than in women with menstrual cycle, for both devices (*P* ≤ 0.035, Mann-Whitney test). Moreover, for women who menstruate within-subject PWV variability was comparable to men (*P* ≥ 0.310).

While the study included 35 participants, we developed multilevel models with 12 repeated measurements per participant for a total of 420 measurements, which considerably increased the study’s power (86). Furthermore, as indicated in the methodology section, the sample size consideration for our model choice allowed for up to 12 independent variables, and we also demonstrated that our estimates of the effects of factors influencing PWV readings were realistic, whenever we could compare our estimates to those estimated in other studies.

One potential limitation of our study was mitigated with the choice of cross-over randomized design. Although we did not directly compare the devices for reasons explained before, it was important that devices be used in a comparable setting as there was a possibility that measurements taken with the first device in a series may affected measurements taken with the second device and that an identified significant factor for a device may be due to the fact that that device is always applied second in the measurement series. To account for this effect, we randomized the order of devices in a measurement series and developed multilevel regression models that showed that the identity of the first device used in a measurement series for a person had no effect on PWV measurements, supporting the assumption of comparable settings.

Finally, because the PWV measurements were taken within 2 weeks, we were unable to investigate any seasonal effects on the PWV measurements. Given that blood pressure can be significantly lower in the summer compared to the winter, it remains to be seen whether the short-term estimates made in this study are sensitive to the season in which the recordings were taken.

## 5. Conclusion

In conclusions, we systematically assessed a large number of experimental, meteorological, physiological factors and personal characteristics to identify those that affect PWV measurements and contribute to differences in short term repeated measurements. We quantified these effects separately for the two devices that use different measurement techniques, using study design that provides strong evidence.

We discovered that increasing age increases not only the values of cfPWV and PWVao but also the variability of their repeated measurements, suggesting that in older people precision of measurement should be increased, possibly with the use of 3–4 measurements in a series instead of only 2.

For SphygmoCor’s cfPWV measurements, we also found a significant interaction between MAP and outdoor temperature, as well as significant mean effect of temperature, which could lead to significant fluctuations in short-term repeated measurements. Recording cfPWV during a season with a higher outdoor temperature (e.g., 25^°^C) when differences in MAP result in smaller differences in cfPWV may reduce some of the short-term fluctuations. Also, it should be further investigated if prolonging resting time during periods with low outdoor temperature might help with reduction of short-term fluctuations.

Time of day or different observers who received the same amount of pre-training did not affect cfPWV or PWVao measurements. However, measurement difficulties occurred significantly less frequently in the afternoon for both instruments. To facilitate future longitudinal studies, it would be advisable that PWV is measured in the afternoon whenever possible, not necessarily with the same observer.

For both devices, we found a relationship between a measurement difficulty and female sex. Whereas, the number of retaken measurements with SphygmoCor was moderately increased in women, the association with sex was very strong for manual signal selection with the Arteriograph device which was much more common in women than in men. In addition, the PWVao measurements by Arteriograph revealed that PWVao values were on average significantly higher in women than in men. This is in contrast to other devices that found higher PWV values for men, or no association with sex. Overall, the findings indicate that devices’ design may be suboptimal for women, with a possible systematic bias in Arteriograph’s measurements.

The differences in factors affecting the PWV measurement in the two models most likely reflect differences in the measurement techniques of the two devices. In addition to age and MAP, cfPWV measurements which are considered a more direct measure of arterial stiffness were also influenced by hypertension status, the interaction MAP-outside temperature, temperature and the order of visits. PWVao values which were estimated from a single site were additionally influenced by patient characteristics such as sex, height, and HR, with higher PWVao values in women being specific to the Arteriograph device.

## Data availability statement

The original contributions presented in this study are included in the article/supplementary material, further inquiries can be directed to the corresponding author.

## Ethics statement

The studies involving human participants were reviewed and approved by the Ethical Committee of University of Split School of Medicine. The patients/participants provided their written informed consent to participate in this study.

## Author contributions

MP and AJ conceived and designed the study, analyzed the data, and wrote the draft of the manuscript. MP, BŠ, AB, PK, VĐ, and IM participated in data collection. MB, IM, and AJ supervised the data collection and the findings of the study. All the authors interpreted the data and approved the final version of the manuscript.
